# Post-pneumonectomy syndrome: a systematic review of the current evidence and treatment options

**DOI:** 10.1186/s13019-023-02278-2

**Published:** 2023-04-10

**Authors:** Natasha Christodoulides, Gerard J. Fitzmaurice, Irmina Bukowska, Eoin O’Rhaillaigh, Conor Toale, Michael Griffin, Karen C. Redmond

**Affiliations:** 1 Department of Thoracic Surgery, The Mater Misericordiae University Hospital, Eccles Street, Dublin, Ireland; 2 Department of Anaesthesia, The Mater Misericordiae University Hospital, Eccles Street, Dublin, Ireland; 3 Royal College of Surgeons in Ireland (RCSI), Dublin, Ireland

**Keywords:** Post-pneumonectomy syndrome, Postpneumonectomy syndrome, Mediastinal repositioning, Prosthesis, Stent

## Abstract

**Objectives:**

Post-pneumonectomy syndrome (PPS) is rare and predominantly characterised by dynamic airway obstruction due to mediastinal rotation at any time point following pneumonectomy. The objective of this systematic review was to identify the optimal treatment strategy for PPS based on subjective symptomatic relief, objective radiological imaging, and treatment durability.

**Methods:**

A systematic review was performed up to and including February 2022 based on the “Preferred Reporting Items for Systematic Reviews and Meta-Analyses” guidelines. All studies that presented the management of symptomatic patients > 16 years of age with radiologically confirmed PPS were included. The primary outcome was the identification of the optimal treatment strategy and the secondary outcome was durability of the treatment. The Oxford Centre for Evidence Based Medicine level was assigned to each study.

**Results:**

A total of 330 papers were identified and reviewed; 41 studies met the inclusion criteria. Data including patient demographics, indication for initial pneumonectomy, presenting symptoms, management approach, outcomes, and follow-up were assessed and analysed. Management approaches were divided into three categories: (a) mediastinal repositioning using implant prostheses; (b) endobronchial stenting; (c) other corrective procedures. One hundred and four patients were identified in total and of those, 87 underwent mediastinal repositioning with insertion of a prosthetic implant. Complications included over- or under-filling of the prosthesis (8.5%) and implant leakage (8.9%).

**Conclusion:**

Management of PPS using a prosthetic implant to reposition the mediastinum is the treatment of choice. Key adjuncts to optimise surgical approach and minimise complications include pre-operative CT volumetric analysis to guide implant size and intra-operative transoesophageal echocardiography to guide mediastinal repositioning.

## Introduction

Post-pneumonectomy syndrome (PPS) is rare and predominantly characterised by dynamic airway obstruction due to mediastinal rotation at any time point following pneumonectomy. For reasons that remain poorly understood, in some patients the mediastinum shifts excessively towards the pneumonectomy space with associated rotation of the great vessels and surrounding structures [1]. This can produce disabling respiratory, cardiovascular, and gastrointestinal symptoms [2].

Progress in determining the optimal management strategy has been slow due to the sparsity of data and lack of long-term follow-up. Reports in the literature suggest benefit from both surgical management, particularly placement of breast prostheses into the pneumonectomy space, but also endobronchial stent placement [2]. There are currently no recommendations and/or consensus guidelines for PPS management from the international thoracic surgical societies.

Consequently, the objectives of this systematic review were to (a) provide an overview of the available evidence, (b) identify the optimal treatment strategy for PPS in terms of subjective symptomatic relief on follow-up, and (c) determine the durability of the treatment method employed based on long-term outcomes.

## Methods

### Search strategy and eligibility criteria

A comprehensive literature search was conducted using MEDLINE, PubMed and EMBASE databases. Search methodology was performed with the assistance of a librarian. The search terms used were (“Post-pneumonectomy syndrome”) OR (“Postpneumonectomy syndrome”). These keywords were selected as they were broad ranging and would identify the maximum number of articles relevant to the chosen topic. The search was limited to the English language and to journal articles only, as these represent the most up-to-date clinical repository. A dedicated search of the “grey literature” (unpublished trials, theses, reports, technical and conference notes) was not undertaken as sufficient contemporaneous data was obtained through the conventional literature search. There were no restrictions to study design and no time limitations up to and including February 2022 (date range: 1979–2022). The abstracts identified in the search were reviewed independently by two reviewers. Any disagreement between reviewers about whether a paper should be included resulted in inclusion at this stage in the process. The full-text articles were then reviewed by two reviewers and a final consensus was reached.

Inclusion criteria were symptomatic patients who had undergone a pneumonectomy with rotation of mediastinal contents on CT-thorax and a clinical diagnosis of PPS. All forms of management were included. The primary outcome was to identify the optimal treatment strategy of PPS, assessed in terms of subjective symptomatic relief on follow-up and/or imaging modalities. The secondary outcome assessed durability of the treatment method used. Literature discussing post-pneumonectomy-like syndrome were excluded. Following assessment of all full-text articles, those discussing the management of PPS in patients < 16 years only were excluded, to avoid confounding factors specific to the paediatric population from affecting the final analysis. In case series and retrospective reviews discussing both paediatric and adult patients, those aged < 16 years old were excluded from the overall analysis (Table 1).

**Table 1:** Inclusion and exclusion criteria

Inclusion criteria	Exclusion criteria
Reports of symptomatic patients with radiological evidence of PPS	Post-pneumonectomy-like syndrome
Journal articles only	Patients < 16 years old
Full text available in English	Patients who did not undergo a corrective procedure

### Data collection

Information was extracted regarding study design, patient numbers, patient age, criteria for initial diagnosis, presenting symptoms, the interval between original and corrective procedures, treatment approach, short- and long-term outcomes. The latter three provided the evidence for our primary and secondary outcomes.

### Analysis of data

**Fig. 1:**
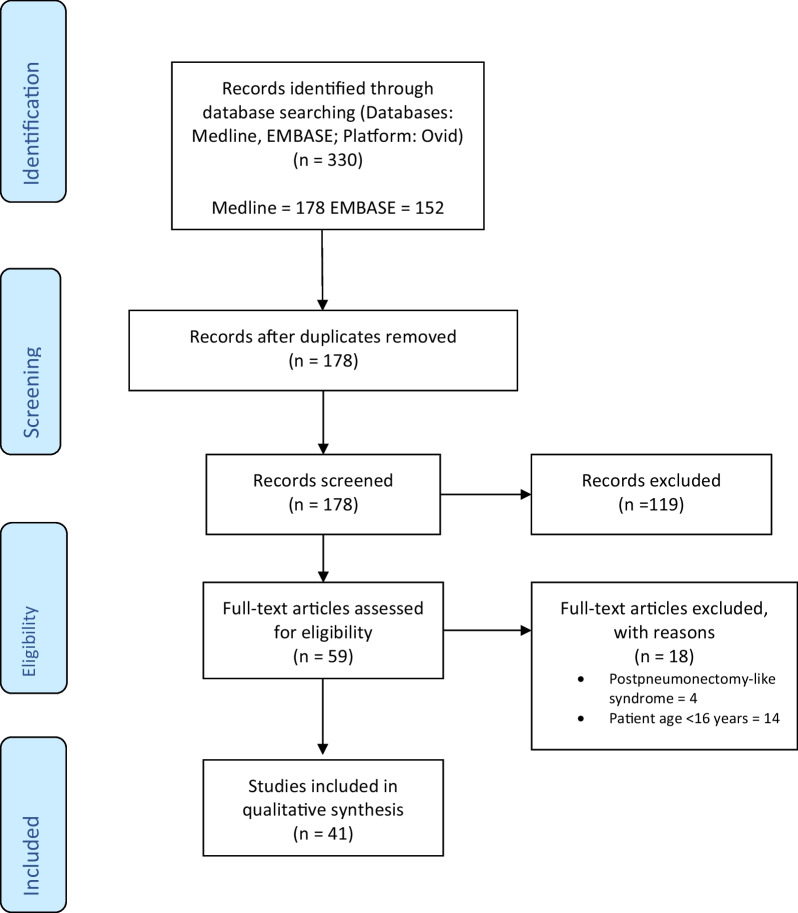
An overview of the literature search strategy with the identification of studies eligible for inclusion in the review

The “Preferred Reporting Items for Systematic Reviews and Meta-Analyses” (PRISMA) guidelines were used to plan and perform this systematic review (Fig. 1). Following the exclusion process, a total of 41 studies were included in the final qualitative synthesis. To assess the level of evidence, an Oxford Centre for Evidence Based Medicine (OCEBM) score [3] was assigned to each study. Data was tabulated and grouped based on the chosen treatment strategy: (a) Prostheses, (b) Endobronchial stenting, (c) Other. This was deemed to be the most comprehensive way to summarise the available research and answer our primary research objective. Data was summarised using descriptive statistics. Where applicable, data was analysed and presented as a proportion of the total. Continuous variables were reported as median values (range). Categorical variables were reported as percentages. Data were analysed using SPSS software (PASW 18.0 for Mac, SPSS Inc., Chicago, IL). Table 2 provides a list of relevant abbreviations and acronyms (Table 2).

**Table 2:** Abbreviations & Acronyms

ARDS	Acute respiratory distress syndrome
CT	Computed tomography
CVP	Central venous pressure
LD	Latissimus dorsi muscle
LRTI	Lower respiratory tract infection
PE	Pulmonary embolism
PPS	Post-pneumonectomy syndrome
PTFE	Polytetrafluoroethylene
TOE	Transoesophageal echocardiogram

## Results

### Study selection and patient demographics

Fourty one studies met the inclusion criteria describing the management and outcome of symptomatic adult patients (> 16 years of age) with radiologically confirmed PPS. There were 31 case reports [4–35], 5 case series [36–40] and 5 retrospective reviews [1, 41–44] that included a total of 113 patients. One patient received treatment for post-pneumonectomy-like syndrome and was excluded from the final analysis. Seven patients were excluded as they were < 16 years of age and one patient was excluded as they did not receive surgical treatment. A total of 104 patients remained for inclusion in the final analysis. Sixty eight patients were female (68.7%) and 31 were male (31.3%). Gender was not reported in one retrospective review that included five patients. PPS developed after a right pneumonectomy in 75 patients (72.1%) and left pneumonectomy in 29 patients (27.9%). Eight patients who developed PPS after a left pneumonectomy had a right-sided aortic arch (27.6%). Sixty-one patients (61.6%) underwent a pneumonectomy for primary lung cancer or pulmonary metastases, while other indications for a pneumonectomy included congenital hypoplasia/bronchial anomaly (10.1%), haemorrhage (7.1%) and pulmonary carcinoid (7.1%) (Table 3). The indication for performing a pneumonectomy was not reported in five patients.

**Table 3:** Patient demographics

Studies, n	41
Patients (total), n	104
Age at time of corrective procedure(s) (years), mean (range) (n = 79)	43.1 (17–75)
Female gender, n (%) (n = 99)	68 (68.7)
*Indication for pneumonectomy (n* = *99)*	
Primary lung cancer/pulmonary metastases, n (%)	61 (61.6)
Congenital hypoplasia/bronchial anomaly, n (%)	10 (10.1)
Haemorrhage, n (%)	7 (7.1)
Pulmonary carcinoid, n (%)	7 (7.1)
Lung abscess, n (%)	2 (2.0)
Bronchiectasis, n (%)	5 (5.1)
Trauma, n (%)	4 (4.0)
Pulmonary sequestration and pulmonary hypertension, n (%)	1 (1.0)
Swyer–James syndrome, n (%)	1 (1.0)
Bronchial stricturing, n (%)	1 (1.0)
*Side of pneumonectomy (n* = *104)*	
Right pneumonectomy, n (%)	75 (72.1)
Left pneumonectomy, n (%)	29 (27.9)
Left pneumonectomy with right aortic arch, n (%)	8 (27.6)

All patients included in the review had varying levels of dyspnoea on presentation, with one patient complaining of positional dyspnoea when lying on their left side [4]. Other common presenting symptoms included audible stridor or wheeze, recurrent respiratory tract infections, persistent cough and symptoms of gastroesophageal reflux. Less frequently reported symptoms included chest pain, haemoptysis, dysphagia and dysphonia.

#### Summary findings of case reports and case series

A total of 36 case reports and case series were included, with 43 patients. The median age at attempted correction was 44.6 years (range 17–75 years). The median interval between the original pneumonectomy and time of attempted correction for PPS was 2.5 years (range 1 month–49 years), with a mean interval of 5.1 years. Seven patients (16.3%) who had undergone a corrective procedure for the treatment of PPS symptoms developed a complication that required repeat intervention. The interval to development of complications following the initial corrective procedure ranged from immediately after extubation to 4 years post-operatively.

#### Summary findings of retrospective reviews

There were five retrospective reviews with a total of 61 eligible patients. The age range at the time of attempted correction was 17–72 years. The overall mortality rate across these studies was 6.6%, with respiratory failure representing the most common cause of death. Recurrence of symptoms or complications requiring repeat intervention was 26.2%.

### Outcomes

The case reports, case series and retrospective reviews included in this study have been divided into three broad approaches to management:

- Mediastinal repositioning with fixed-volume prostheses/tissue expanders;
- Endobronchial stenting to relieve bronchial stenosis/obstruction;
- Other corrective procedures.

Short- and long-term outcomes relating to the three broad management approaches are outlined in Tables 4, 5 and 6 respectively. Short-term outcomes included any complications within 30 days of the corrective procedure.

**Table 4:** Outcomes (prostheses)

Study	n	Prosthesis (no.) (total volume, mls)	Implant sizing	Interval between original and corrective surgery	Short-term outcome (no. of patients)	Length of follow-up	Long-term outcome (no. of patients)	Surgical revision of prosthesis (Y/N) (no. of patients)	OCEBM score
Avgerinos et al. [18]	1	Tissue expander (1) (475mls)	Slow filling of tissue expander while monitoring CVP	6 y	Good	6 mo	Good	N	4
Gebitekin and Bayram [8]	1	Tissue expander (1) (600mls)	–	3 y	Recurrence of symptoms: overfilling	5 y	Good	N	4
Matheson et al. [6]	1	Tissue expander (2) (490mls)	TOE monitoring	4 y	Good	3 mo	Good	N	4
Bobbio et al. [9]	1	Tissue expander (1) (700mls)	–	18 mo	Mild ongoing symptoms: underfilling	5 mo	Good	N	4
Codsi et al. [19]	1	Fixed volume (1) (300mls) Tissue expander (1) (50mls)	Intra-operative bronchoscopy	3 y	Re-intubation: hypercapnia (9d)	1 y	Good	N	4
Boiselle et al. [20]	1	Fixed volume (1)	–	14 y	–	–	–	N	4
Mahesh et al. [7]	1	Fixed volume (2) (1310mls)	Silicone prosthesis sizers	4 y	Good	–	–	N	4
Partington et al. [4]	1	Fixed volume (2)	TOE monitoring	6 mo	Good	–	–	N	4
Jansen et al. [21]	2	Fixed volume (1) (150mls) Fixed volume (1) (1900mls)	CVP monitoring	6 mo 1 mo	Good (2)	20 mo –	Good (2)	N (2)	4
Audry et al. [39]	2	Fixed volume (1) (360mls) Fixed volume (1) (300mls)	–	8 y 10 y	Good (2)	3 y 1 y	Ongoing dyspnoea (1) Good (1)	Y: Leak (1) N (1)	4
Bedard et al. [36]	3	Fixed volume (2) (1300mls) Fixed volume (1) (1950mls) Fixed volume (1) (1700mls)	–	3 y 1 y 5 wk	Good (2) Dyspnoea: resolved without further intervention (1)	–	Good (2) Not reported (1)	N (3)	4
Reed and Lewis [22]	1	Fixed volume (2) (1150mls) Thoracoscopic approach	Intra-operative bronchoscopy	7 mo	Good	3 mo	Good	N	4
Bastin et al. [23]	1	Fixed volume (1)	Saline instillation into the hemithorax	6 wk	Good	1 mo	Good	N	4
Kelly et al. [11]	1	Fixed volume (2) (1,300mls)	Intra-operative bronchoscopy	6 y	Modest symptomatic relief	1 y	Good	N: residual bronchomalacia, metallic stent insertion	4
McRae et al. [10]	1	Fixed volume (2) (960mls) Alloderm sheet	–	19 y	Good	2 y	Good	Y: rupture; insertion of custom-made implant with thick wall	4
Ng et al. [24]	1	Fixed volume (2) (1220 ml) Thoracoscopic approach	Intraoperative bronchoscopy; CVP monitoring	7 mo	Good	6 mo	Good	N	4
Riveron et al. [25]	1	Fixed volume (2) (1010 ml)	–	2 y	Good	–	Good	N	4
Tsunezuka et al. [26]	1	Custom-made tissue expander (1) (600 ml)	Preoperative CT volumetric analysis	3 y	Good	1 y	Good	N	4
Sharifpour and Bittner [27]	1	Fixed volume (1)	–	10 mo	–	–	–	–	4
Wang et al. [5]	1	Custom-made 3D carbon fibre-printed (1) Teflon patches	Preoperative CT volumetric analysis and 3D electron modelling	2 y	Good	1 y	Good	N	4
Encarnacion et al. [28]	1	Fixed volume (1) Tissue expander (1)	Preoperative CT volumetric analysis	2 y	Good	6 mo	Good	N	4
Birdi et al. [29]	1	Fixed volume (3) Pericardiopexy	–	6 y	Good	6 y	Good	N	4
Smulders et al. [34]	1	Fixed volume (2)	–	6 y	Good	6 mo	Good	N	4
Manna et al. [33]	1	Fixed volume (4) (1160 ml) Pericardial and intercostal flap	–	3 mo	Good	3 mo	Good	N	4
Shen et al. [1]	17	17 pts: Fixed volume (mean: 900 ml) 9 pts: Pericardiopexy	Saline instillation into the hemithorax; intraoperative bronchoscopy; CVP monitoring	Median: 7.7 y Range: 1.1–54.8 y	Pneumonia (3) ARDS (2) Died post-op multi-system organ failure (1) Overfilling (4) Good (7)	Median: 32 mo Range: 4–143 mo	Good (16) Died 4 y post-op: airway malacia and symptom recurrence (1)	Y: symptom recurrence & removal of prosthesis 1 y post-operatively (1) N (15)	4
Grillo et al. [37]	8	8 pts: Fixed volume (mean: 911 ml) 2 pts: Pericardiopexy	HR and BP monitoring	Range: 5 mo–17 y	Good (5) Mild pericardial compression then good (1) Improved (1) Died—respiratory failure (1) Died 1 mo post-op—PE (1)	Range: 1 mo–6 y	Symptom recurrence 4 y post-op (1) Good (6)	N (8)	4
Podevin et al. [43]	2	2 pts: Tissue expander (mean: 385 ml)	–	8.3 y 7.5 y	Good (2)	4.5 y 4 y	Good (1) Moderate (1)	Y: Leak (2)	4
Macare Van Maurik et al. [41]	19	19 pts: Tissue expander (range: 200–1750 ml)	Intraoperative bronchoscopy; CVP monitoring	Range: 4 mo–15 y	Good (17) Luxation (1) Malposition (1)	Range: 3 mo–5 y	Good (15) Herniation (1) Leak (3)	Y: Herniation (1), leak (3), luxation (1), malposition (1) N (13)	4
Valji et al. [44]	5	5 pts: Fixed volume 5 pts: Pericardiopexy	Intraoperative bronchoscopy; CVP monitoring	Median: 21 mo Range: 9 mo–29 y	Good (5)	Range: 7 mo–6 y	Good (5)	N (5)	4
Soll et al. [42]	5	1 pt: Tissue expander 4 pts: Fixed volume Median: 945mls (range: 200–1950mls)	CVP monitoring	Median: 30 mo Range: 10 mo–8 y	Good (5)	4 y	Good (3) Leak of tissue expander (1) Recurrent LRTIs (1)	Y: Leak (1) N (4)	4
Shamji et al. [40]	3	3 pts: Fixed volume (range: 900–1600 ml)	Intraoperative bronchoscopy	Median: 3 mo Range: weeks–6 mo	Good (2) SVT in immediate post-operative period (1)	Range: 1.5–3 y	Good (3)	Y: SVT due to overcorrection of mediastinal shift and impaired atrial filling. Corrected by reducing volume (1) N (2)	4

*d* Days; *mo* Months; *y* Years

OCEMB Score: 1a—SR of RCTs; 1b—Individual RCT; 1c—All or none; 2a—SR of cohort studies; 2b—Individual cohort study; 2c—“Outcomes” research; 3a—SR of case–control studies; 3b—Individual case–control study; 4—Case series; 5—Expert opinion

**Table 5:** Outcomes (Endobronchial stenting)

Study	n	Procedure (no. of patients)	Interval between original and corrective surgery	Short-term outcome (no. of patients)	Length of follow-up	Long-term outcome (no. of patients)	Revision (Y/N) (no. of patients)	OCEBM score
Harney et al. [12]	1	Metallic stent	49 y	LRTI	30 mo	Good	Y: re-stenting	4
Moser et al. [14]	1	Silastic stent	12 y	Good	5 mo	Died: alcohol intoxication and aspiration	N	4
Abe et al. [17]	1	2 metallic stents	3 y	Good	12 y	Recurrent LRTIs. Died—respiratory failure	N	4
Nakamura et al. [13]	1	Balloon dilation	10 y	Poor; stent inserted	3 mo	Died–MI	N	4
Cordova et al. [30]	1	2 metallic stents	4 y	Good	1 y	Good	N	4
Stratakos et al. [38]	1	Metallic stent	2 y	Poor	6 mo	Died–PE	Y: silastic stent insertion	4

*mo* Months; *y* Years

OCEMB Score: 1a—SR of RCTs; 1b—Individual RCT; 1c—All or none; 2a—SR of cohort studies; 2b—Individual cohort study; 2c—“Outcomes” research; 3a—SR of case–control studies; 3b—Individual case–control study; 4—Case series; 5—Expert opinion

**Table 6:** Outcomes (other corrective procedures)

Study	n	Procedure (no. of patients)	Interval between original and corrective surgery	Short-term outcome (no. of patients)	Length of follow-up	Long-term outcome (no. of patients)	Revision (Y/N) (no. of patients)	OCEBM score
Chujo et al. [15]	1	Mediastinal fixation with PTFE sheet	2 y	Good	6 mo	Died—cancer recurrence	N	4
Soll et al. [42]	1	Mediastinal fixation with a xenopericardial graft (1)	Median: 30 mo Range: 10 mo–8 y	Good	4 y	Graft infection	Y: removal of pericardial patch	4
Wasserman et al. [31]	1	Thoracotomy and release of adhesions (no prosthesis)	1 y	Poor	6 mo	Good	Y: repositioning with prostheses (990mls) sutured to chest wall	4
Grillo et al. [37]	4	Aortic division and bypass to relieve bronchial compression (1) Reposition (no prosthesis) (3)	Range: 5 mo–17 y	Died—pneumonia, bronchomalacia (1) Reposition without prosthesis failed (3)	Range: 1 mo–6 y	Died—respiratory failure (1) Good (2)	Y: re-operations required (3)	4
Jansen et al. [21]	1	Chemical blockage of phrenic nerve	6 mo	Poor	20 mo	Good following re-positioning with tissue expander	Y	4
Karasaki et al. [32]	1	Emergency window thoracostomy; LD muscle flap	3 mo	Good	2 mo	Good	N	4
Uyama et al. [16]	1	Intra-pleural injection of Sulpur hexafluoride (SF_6_)	18 mo	Good	SF_6_ injections every 6 mo	Good	N	4
Shamji et al. [40]	1	Resection of a portion of the adjacent thoracic vertebra	6 y	Good	4 mo	Died—cause unknown	N	4
Gullung and Halstead [35]	1	Vocal fold medialisation with Radiesse^®^ voice gel injection	6 y	Poor—unsatisfactory subjective improvement of dysphonia	Lost to follow-up	–	N	4

*mo* Months; *y* Years

OCEMB Score: 1a—SR of RCTs; 1b—Individual RCT; 1c—All or none; 2a—SR of cohort studies; 2b—Individual cohort study; 2c—“Outcomes” research; 3a—SR of case–control studies; 3b—Individual case–control study; 4—Case series; 5—Expert opinion

#### Prostheses: safety and durability

The most common initial management method for PPS was mediastinal repositioning with fixed-volume prostheses or tissue expanders (78.6% of studies including a total of 87 patients) (Table 4). Saline-filled tissue expanders were used either alone or in combination with fixed-volume prostheses in 28 patients (32.2%), allowing percutaneous volume adjustment. Wang et al. described the use of a 3D carbon fibre-printed implant with good results [5]. Sizing of prostheses was reported in 18 studies with perioperative CVP monitoring (n = 7) and bronchoscopic assessment of airway compression (n = 8) being the most common approaches. Pre-operative CT volumetric analysis (n = 3) and intra-operative instillation of saline into the empty hemithorax (n = 3) were also described. Two studies reported the use of perioperative TOE monitoring [4, 6], while one case report described the use of silicone prosthetic sizers that were then replaced by fixed-volume prostheses [7] (Table 4).

Sixty four out of 85 patients (75.3%) had no reported complications in the short-term. Two studies did not comment on short-term outcomes. Within 4 weeks of surgery, 9.4% of patients (n = 8) presented with symptom recurrence secondary to over- or under-filling of the prosthesis [1, 8, 9, 36, 40]. The implant volume was adjusted percutaneously in 2 patients, one patient required repeat thoracotomy 5 days after the initial procedure [40], while one patient’s symptoms resolved without intervention [36]. Four patients showed evidence of haemodynamic compromise intra-operatively, requiring reopening of their partially closed incision and reduction of the prosthetic volume [1]. Macare Van Maurik et al. reported short-term complications in 2 patients; namely implant luxation 2 weeks after repositioning and malposition 3 weeks after repositioning [41]. Three patients died within one month of the corrective procedure (massive PE, multi-system organ failure, and respiratory failure) [1, 37].

Nine out of thirty-three studies did not report individual figures for length of follow-up. Of the remaining studies, median length of follow-up was 1 year (range 1 month–6 years). Long-term outcome was reported in 82 patients, with 13 patients (15.9%) requiring repeat surgical intervention. The most common long-term complication requiring surgical revision was leakage of the prosthesis (8.9%). Following implant leakage, McRae et al. reported the use of a custom-made implant with a wall three times thicker than the initial prosthesis with good results [10]. Shen et al. described a case of re-do thoracotomy at 1 year post-operatively to remove the prosthesis due to symptom recurrence [1]. The same study reported one patient who died 4 years post-operatively due to symptom recurrence secondary to airway malacia [1]. In their case report, Kelly et al., described insertion of an endobronchial stent to relieve ongoing dyspnoea due to recurrent airway malacia after the insertion of a prostheses with good outcomes [11] (Table 4).

#### Endobronchial stenting: safety and durability

Endobronchial stenting as the primary management method for PPS was described in six studies (n = 6) (Table 5). Metallic stents (n = 4) were associated with a 50% short-term complication rate, with reports of stent migration, partial obstruction and recurrent LRTIs [12, 38]. Two of these patients required repeat stenting for symptomatic relief. Median length of follow-up was 9 months (range 3 months–12 years). Four of the 6 patients died during the follow-up period; one died of a PE and the other of respiratory failure 6 months and 12 years later, respectively [37, 38]. Nakamura et al. reported a case of endobronchial stenting following failed endobronchial balloon dilation with limited follow-up [13]. Moser et al. described the successful use of a silastic stent with limited follow-up (n = 1) [14] (Table 5).

#### Other corrective procedures

Other reported corrective procedures of PPS included mediastinal graft fixation (n = 2), repositioning the mediastinum (without prostheses) (n = 4), chemical blockage of the phrenic nerve (n = 1), intra-pleural injection of sulphur hexafluoride (SF_6_) (n = 1) and resection of a portion of the adjacent thoracic vertebra (n = 1) (Table 6). The use of bioprosthetic patches and synthetic meshes to maintain the mediastinal position have shown varying results and insufficient follow-up [15, 42]. Uyama et al. described the echocardiography-guided injection of SF_6_ into the pleural space in a high-risk surgical candidate with satisfactory symptomatic improvement [16]. Gullung and Halstead describe a case of dysphonia (diminished vocal volume) 6 months following a left pneumonectomy, as a result of right recurrent laryngeal nerve compression due to PPS. This was treated with Radiesse^®^ voice gel injection, without satisfactory subjective improvement [35]. Overall outcomes following repositioning without prostheses were poor, requiring an additional procedure in all cases [16, 17].

## Discussion

### Summary

Post-pneumonectomy syndrome is a rare condition that involves rotation of the mediastinum towards the contralateral hemi-thorax at any time point following pneumonectomy [1]. This results in extrinsic compression of the trachea or bronchus against the descending thoracic aorta and/or spine due to mediastinal shift with herniation and over-inflation of the remaining lung parenchyma [32]. Symptoms that result may take a progressive or indolent course with the potential for disabling long-term outcomes if left untreated [37]. It is therefore important to maintain a high index of suspicion in patients who have previously undergone a pneumonectomy.

A number of methods to attempt surgical correction for PPS have been reported, consisting mostly of single case reports and case series. In this review we have outlined the current evidence and potential treatment options. We sought to determine the optimal treatment strategy assessed in terms of subjective symptomatic relief and durability.

PPS occurred in 72.1% of patients after a right pneumonectomy. Amongst patients with a left pneumonectomy, 27.6% had a right-sided aortic arch, indicating the importance of site and underlying anatomy in risk stratification. Surgical correction had a significant overall mortality risk of 6.5% (n = 6) and a recurrence rate of between 16.9 and 26%. Risk factors for symptom recurrence included sub-optimal prosthetic implant sizing and implant leakage, indicating the importance of optimising implant choice and size as well as mitigating implant trauma from surrounding tissue at the time of surgical correction. Surgical management without a prosthetic implant had a 100% incidence of recurrence and should be avoided. Endobronchial stents had a 50% complication rate, albeit with small and high-risk patient numbers. Consequently, optimal management of these complex cases is challenging, however surgical management with a prosthetic implant in suitable patients under appropriate image guidance appears to offer the safest and most durable treatment strategy.

### Method of mediastinal repositioning

We have identified 42 studies discussing the management of PPS in adult patients (> 16 years of age). The literature favours mediastinal repositioning and placement of implant prostheses, using either open or thoracoscopic approaches [22, 24]. Luxation and malposition are two reported implant-specific short-term complications requiring repeat thoracotomy [41]. The most common long-term complication is implant leakage (8.9%). The exposed bronchial stump staple line can be a source of trauma to the prosthesis, resulting in leakage and symptom recurrence [24]. The use of prophylactic AlloDerm did not protect the implants from trauma [10, 24]. McRae et al. described the successful use of a custom-made tissue expander with a wall three times as thick as normal implants [10]. Custom made, patient-specific prostheses, as well as 3D carbon-fibre printed prostheses, have also been described with good results [5, 26]. In cases where patients develop severe airway malacia following mediastinal repositioning, airway stenting may relieve symptoms, as described by Kelly et al. [11]. Overall mortality in the patient cohort managed with prostheses was 4.8%.

Other corrective procedures described include the use of bioprosthetic patches and synthetic meshes to maintain mediastinal position. The use of a xenopericardial graft described by Soll et al. required removal 5 months later due to infection [42]. Chujo et al. used a PTFE mesh to re-position the mediastinum through a right mini-thoracotomy with uncertain long-term effects as the patient died of cancer recurrence 6 months later [15].

### Complications of non-surgical management

Endobronchial stenting with either metallic or silastic stents was described in six case reports (n = 6). These patients had multiple co-morbidities rendering surgical mediastinal repositioning inappropriate. Overall, use of metallic stents alone had unfavourable outcomes, including stent migration, obstruction, residual narrowing and recurrent LRTIs [32, 38]. Bronchial stenting with a silastic stent alone has been described with good results in one patient, despite insufficient follow-up [24]. Overall mortality in this patient cohort is 66.7%, with one patient dying of respiratory failure 12 years post-nitinol stent insertion following recurrent hospitalisations with LRTIs [17]. Stenting would be inappropriate in branching, distorted bronchi due to difficulty in stent placement and poor stent epithelialisation [30]. It is therefore important to take an individualised approach when considering the optimal treatment method in co-morbid patients with PPS.

### Limitations

A meta-analysis was not feasible due to the heterogeneity between the studies. Many studies did not satisfy the secondary outcome i.e. durability of treatment method used, as the follow-up period was not explicitly stated or insufficient. As a result, it was difficult to compare long-term outcomes. Moreover, the patients who had endobronchial stenting as the primary management method for PPS likely had more co-morbidities and therefore worse outcomes, making a comparison with other treatment methods biased.

The indication for a pneumonectomy was not taken into consideration when calculating survival outcomes. In particular, the initial tumour stage was not taken into account in patients with primary lung cancer who later developed PPS. This could affect survival outcomes and is therefore a limitation of this study.

An additional problem was that some retrospective reviews and case series incorporated individual patients that did not satisfy the inclusion criteria for the study. These patients were therefore excluded from our data analysis. Consequently, any results or conclusions made in these studies may not be reflected in this systematic review.

## Conclusion

PPS is a challenging clinical scenario. This study represents, to our knowledge, the most complete review highlighting the current evidence and treatment options in patients > 16 years of age. Post-pneumonectomy syndrome managed without a prosthetic implant had a 100% incidence of recurrence and high complication rate. Mediastinal repositioning using a prosthetic implant demonstrates the most favourable outcome in terms of anatomical realignment, symptomatic relief, and overall durability. Sizing of the prosthetic implant can be achieved with the use of CT volumetric analysis and peri-operative TOE guidance.

## Data Availability

The datasets generated and/or analysed during the current study are available from the corresponding author on request.
